# Evaluation of hematological alteration of vector‐borne pathogens in cats from Bangkok, Thailand

**DOI:** 10.1186/s12917-020-02737-1

**Published:** 2021-01-18

**Authors:** Thom Do, Ketsarin Kamyingkird, Wissanuwat Chimnoi, Tawin Inpankaew

**Affiliations:** Department of Parasitology, Faculty of Veterinary Medicine, Kasetsart University, 10900 Bangkok, Thailand

**Keywords:** Vector-borne pathogens, *Babesia*, *Hepatozoon*, Hemoplasmas, Hematological parameters, Semi-domesticated cats

## Abstract

**Background:**

Cats can be carriers of infected arthropods and be infected with several vector-borne pathogens (VBPs) but there is limited knowledge about their pathogenic role in cats. This study aimed to assess the prevalence of some feline vector-borne agents by molecular technique and to characterize the hematological findings associated with these infections in a cat population from Bangkok Thailand.

**Results:**

PCR was positive with at least one pathogen in 237 out of 372 subjects (63.7%), with prevalence of 39.5% (147/372) for *Babesia* spp., 36.9% (137/372) for hemoplasmas and 3.2% (12/372) for *Hepatozoon* spp. The cats older than 1 year were at significantly greater risk for VBPs infection (*P* = 0.001; OR = 1.43; 95% CI: 1.12 – 1.81) and hemoplasmas infection (χ2 = 10.8, df = 1; *P* < 0.0001; OR = 2.45; 95% CI: 1.49 – 4.01). A significant association between hematological findings and hemoplasma infection were identified in the present study. Besides, VBPs infection revealed more frequent in male cats (χ2= 6.38, df = 1, *P* = 0.01). Macrocytic hypochromic type of anemia was observed in cats infested with blood-sucking arthropods compared to the non-infested cats presented.

**Conclusions:**

The current study confirmed that *Babesia*, *Hepatozoon* and hemoplasmas had infected semi-domesticated cats in Bangkok, Thailand, with *Babesia* and hemoplasmas being dominant in prevalence. Some hematological findings were significantly associated with cats infected with vector-borne pathogens in this study including leukocyte count and platelets count that may help support veterinary technicians in diagnosis and appropriate treatment. Campaigns of VBPs monitoring in Bangkok emphasizing on the investigation of vectors and possible routes of the infection in animals should be conducted to prevent the transmission of the pathogens.

## Background

Vector-borne pathogens (VBPs) can cause significant impact on the health status of various animals, human included, which are transmitted by a variety of vectors, such as ticks, flies, mosquitoes, fleas and etc. [1]. The most common vector-borne diseases in animals worldwide (predominantly in dogs and cats) are babesiosis, hepatozoonosis and hemoplasmosis which are caused by *Babesia* spp., *Hepatozoon* spp. and hemotropic mycoplasma, respectively [1]. *Babesia* and *Hepatozoon*, tick-borne intracellular protozoan parasites and the hemotropic mycoplasma (hemoplasmas), a group of uncultivatable bacteria within the genus *Mycoplasma* are known as important causes of emerging and re-emerging illness in cats and can induce variable degrees of hemolytic anemia [2, 3]. The clinical signs associated with these pathogens are often vague or non-specific and seem to be associated with fever, lethargy and anorexia in most cases [4].

Hematological alterations are indicators of several vector borne diseases and has been considered to be good tools to support practitioner for clinical diagnosis of VBPs infection which could reveal a wide range of different types of anemia in symptom [5, 6]. Other hematological abnormalities such as leukopenia, leukocytosis and thrombocytopenia, have been sporadically documented in vector-borne pathogen infections in animals [6, 7]. However, the association between these hematological parameters and vector-borne infection in cats in Thailand are limited.

In order to identify the specific pathogens in an infected animal currently, molecular testing has shown advantages of high specificity and sensitivity in being able to accurately identify and differentiate VBPs from others [4]. Research developments regarding molecular techniques in diagnosis have been rapidly applied in epidemiological investigation and have shown that the prevalence of VBPs based on *Babesia, Hepatozoon* and hemoplasmas in cats varies worldwide [4, 8–13].

In Bangkok, a myriad number of stray cats and dogs are allowed roaming the streets, public places and Buddhist monastery [14]. These animals were reported with a high prevalence (90.0%) of flea, tick and lice infection and indicated to be potential sources for some vector-borne diseases [15, 16]. Many communities in Thailand, owned cats are allowed to roam freely outdoors. Such a status can create a risk for human health, when they might contact with infected animals outside and bring infection into the home and transmit it directly to human. This study aimed to assess the prevalence of some feline vector-borne agents by molecular technique and to characterize hematological abnormalities associated with these infections in a cat population from central Thailand to provide additional support for veterinary technicians and veterinarians in diagnosis and treatment.

## Results

### VBPs infection in cats

Out of the 372 semi-domesticated cats, the overall prevalence of VBPs infection was 63.8% (237/372). The prevalence of *Babesia* spp., *Hepatozoon* spp. and hemoplasmic DNA were 39.5% (147/372), 3.2% (12/372) and 36.9% (137/372), respectively. The details were presented in Table 1. Risk factor analysis of VBPs infection in cats showed that, the cats older than 1 year were at significantly greater risk for VBPs infection (*P* = 0.001; OR = 1.43; 95% CI: 1.12–1.81) and hemoplasmas infection (χ^2^ = 10.8, *df* = 1; *P <* 0.0001; OR = 2.45; 95% CI: 1.49–4.01). Similarly, the same result was also observed in cats older than 1 year for *Babesia* spp. and hemoplasmas co-infection (χ^2^ = 5.53, *df* = 1, *P* = 0.01). However, this was not the case for *Babesia* spp. (χ^2^ = 0.81, *df* = 1, *P* = 0.36) or *Hepatozoon* spp. (χ^2^ = 0.006, *df* = 1, *P* = 0.936) infection alone (Table 1). Some statistically significant associations between cat gender (male 46.0%, female 54.0%) and vector-borne prevalence were detected. Specifically, male cats were more likely to be infected with any VBPs (χ^2^ = 6.38, *df* = 1, *P* = 0.01) and with hemoplasmas (χ^2^ = 7.29, *df* = 1, *P* = 0.006) compared to female cats. Male cats were 1.64 times (95% CI: 1.06–2.54) and 1.84 times (95% CI: 1.20–2.81) at higher risk of infection from any VBPs and hemoplasmas, respectively (Table 1). Cats were visually inspected for ectoparasite infestation (50.5%, 188/372). The species of ectoparasite presented in the study included *Rhipicephalus sanguineus, Ctenocephalides felis felis and Felicola subrostratus.* However, no significant differences in vector-borne prevalence were detected between ectoparasite infestations and non-ectoparasite infestations.

**Table 1:** Distribution of vector-borne infection in semi-domesticated cats based on epidemiological data

Variable	Total number	Number of negative cats (%)	Number of positive cats (%)							
			**Any pathogen**	***Babesia*** **spp.**	***Hepatozoon*** **spp.**	**Hemoplasmas**	**BB + HM**	**BB + HP**	**HM + HP**	**BB + HM + HP**
	372	135 (36.3)	237 (63.8)	147 (39.5)	12 (3.2)	137 (36.9)	50 (13.4)	3 (0.8)	6 (1.6)	2 (0.5)
**Age**										
Young (≤ 1-year-old)	81 (21.8)	42 (51.9)	39 (48.1)	13 (16.1)	2 (2.5)	14 (17.2)	4 (4.9)	0	1 (1.2)	0
Adult (> 1-year-old)	291 (78.2)	93 (32)	198 (68)*	134 (46.1)	10 (3.4)	123 (42.3)*	46 (15.9)*	3 (1.0)	5 (1.7)	2 (0.7)
**Gender**										
Male	171 (46)	49 (28.7)	122 (71.3)*	73 (42.7)	6 (3.5)	76 (44.4)*	29 (17)	2 (1.2)	3 (1.8)	1 (0.6)
Female	201 (54)	86 (42.8)	115 (57.2)	74 (37)	6 (3)	61 (30.3)	21 (10.4)	1 (0.5)	3 (1.5)	1 (0.5)
**Ectoparasite infestation**										
Yes	188 (50.5)	67 (35.6)	121 (64.4)	82 (44)	10 (5.3)	65 (35)	28 (14.9)	3 (1.6)	5 (2.7)	2 (1.0)
No	184 (49.5)	68 (37)	116 (63)	65 (35.3)	2 (1.1)	72 (39.1)	22 (12)	0 (0.0)	1 (0.5)	0 (0.0)
**Tick infestation**										
Yes	7 (1.9)	4 (57.1)	3 (42.9)	1 (14.3)	0	3 (42.9)	1 (14.3)	0	0	0
No	365 (98.1)	247 (67.7)	118 (32.3)	81 (22.2)	10 (2.7)	62 (17)	27 (7.4)	3 (0.8)	5 (1.4)	2 (0.5)
**Louse infestation**										
Yes	8 (2.2)	4 (50)	4 (50)	3 (37.5)	0	2 (25)	1 (12.5)	0	0	0
No	364 (97.8)	247 (67.9)	117 (32.1)	79 (21.7)	10 (2.7)	63 (17.3)	27 (7.4)	3 (0.8)	5 (1.4)	2 (0.5)
**Flea infestation**										
Yes	179 (48.1)	69 (38.5)	110 (61.5)	73 (40.8)	10 (5.6)	58 (32.4)	25 (14)	3 (1.7)	5 (2.8)	2 (1.1)
No	193 (51.9)	182 (94.3)	11 (5.7)	9 (4.7)	0	7 (3.6)	3 (1.6)	0	0	0

Abbreviations: *BB* *Babesia* spp, *HM *Hemoplasmas, *HP* *Hepatozoon* spp

*Statistically significant differences (*P* < 0.05)

### Hematological findings

In the current study, the majority of hematological findings of control groups have fallen between the normal ranges [15], except for WBC. The hematological findings revealed differences between the infected and control groups of cats in the WBC and PLTs parameters (Table 2). Specifically, there was a significant reduction (*P* = 0.04, 95% CI: 0.02–3.35) in the number of leukocytes (18.12 ± 6.9) and a significant decrease in the number of thrombocytes (394 ± 148.4) of hemoplasmas-infected cats compared to non-infected cats (19.68 ± 8.7; 438 ± 169.3), respectively. A low number of thrombocytes were observed between the VBP-infected group (401.7 ± 146; *P =* 0.03, 95% CI: 2.75–71.54) and negative group (438 ± 169.3) but not infection alone with *Babesia* spp. (*P =* 0.33, 95% CI: -16.21–47.5) or *Hepatozoon* spp. (*P =* 0.28, 95% CI: -165.37–54.02). Some additional significant associations were detected between these hematological data and ectoparasite-infested prevalence in cats. Cats infested with ectoparasites showed a significant difference compared to non-infested cats regarding erythrocyte, hemoglobin value, MCV and MCHC values. Ectoparasite-infested cats showed macrocytic hypochromic anemia through an increase in the MCV (49.21 ± 5.3) and a decline in the MCHC (31.42 ± 2.2) compared to the non-infested group (*P* < 0.001, 95% CI: 0.29–1.12). Some other hematological abnormalities determined in cats infested with ectoparasites were a low number of erythrocytes (RBCs) (*P* = 0.04, 95% CI: 0.004–0.59) and a low concentration of hemoglobin (HGB) (*P* = 0.0233, 95% CI: 0.06–0.93) (Table 2).

**Table 2:** Selected hematological findings on vector-borne pathogens PCR-positive cats and PCR-negative cats and of ectoparasitic infestation and ectoparasitic non-infestation in cats

Hematological parameters	Negative cats – Mean (± SD) (133)	Positive cats (total number) - Mean (± SD)				Ectoparasite- Mean (± SD)	
		**Any pathogen** **(237)**	***Babesia*** **spp.** **(147)**	***Hepatozoon*** **spp. (12)**	**Hemoplasmas (137)**	**Non-infestation** **(184)**	**Infestation** **(188)**
WBC (× 10^3^/µl)	19.68 (± 8.7)	18.92 (± 7.4)	19.25 (± 7.7)	19.57 (± 5.5)	18.12 (± 6.9)**	19.04 (± 8.4)	19.32 (± 7.4)
RBC (× 10^6^/µl)	7.4 (± 1.4)	7.576 (± 1.5)	7.7 (± 1.4)	7.1 (± 1.7)	7.38 (± 1.6)	7.69 (± 1.4)	7.39 (± 1.6)**
HGB (g/dl)	11.46 (± 2.1)	11.62 (± 2.1)	11.7 (± 2.1)	11.4 (± 2.3)	11.4 (± 2.3)	11.81 (± 2)	11.31 (2.2)**
HCT (%)	36 (± 6.5)	36.72 (± 6.8)	36.9 (± 6.4)	35.15 (± 6.7)	36.3 (± 7.2)	36.9 (± 6.3)	36.02 (± 7.0)
MCV (fl.)	48.43 (± 4.5)	48.81 (± 5.1)	48.7 (± 5.2)	49.1 (± 3.8)	49.5 (± 5.3)	48.11 (± 4.4)	49.21 (± 5.3)**
MCH (pg)	15.38 (± 1.1)	15.42 (± 1.2)	15.4 (± 1.2)	15.9 (± 1.1)	15.5 (± 1.2)	15.41 (± 1.0)	15.39 (± 1.24)
MCHC (g/dl)	31.88 (± 2.2)	31.72 (± 2.3)	31.7 (± 2.1)	32.4 (± 0.9)	31.5 (± 2)	32.13 (± 1.9)	31.42 (± 2.2)**
PLT (× 10^3^/µl)	438 (± 169.3)	401.7 (± 146)**	405.5 (± 147.1)	468.8 (± 171.4)	394 (± 148.4)**	412.58 (± 149.3)	417.27 (± 161.81)

Abbreviations: *WBC *white blood cell; *RBC *red blood cell; *HGB *hemoglobin; *HCT* hematocrit; *MCV* mean corpuscular volume; *MCH *mean corpuscular hemoglobin; *MCHC* mean corpuscular hemoglobin concentration; *PLT* platelets

**Statistically significant differences (*P* < 0.05)

The study found that cats infected with at least one VBP represented 58.9% (33/56) for anemia, 100% (3/3) for leukopenia, 63.6% (178/280) for leukocytosis and 63.6% (61/88) for thrombocytopenia. Hemoplasmas infection (41.0%; 23/56) was most frequent in anemic cats, followed by *Babesia* infection (32.1%; 18/56) and *Hepatozoon* infection (3.6%; 2/56). However, no significant associations were found between CBC abnormalities and vector-borne PCR-positive results (Table 3).

**Table 3:** Different hematological findings of vector-borne infection in semi-domesticated cats

Hematology	Total number of cats	Number of negative cats (%)	Number of positive cats (%)							
			**Any pathogen**	***Babesia*** **spp.**	**Hemoplasmas**	***Hepatozoon*** **spp.**	**BB + HM**	**BB + HP**	**HM + HP**	**BB + HM + HP**
	372	135 (36.3)	237 (63.8)	147 (39.5)	137 (36.8)	12 (3.2)	50 (13.4)	3 (0.8)	6 (1.6)	2 (0.5)
**RBC (× 10**^**6**^**µl)**										
High (> 10)	16 (4.3)	5 (31)	11 (69)	8 (50)	4 (25)	0	1 (6.2)	0	0	0
Normal (5–10)	339 (91.1)	124 (36.6)	215 (63.4)	134 (39.5)	125 (36.9)	11 (3.2)	47 (13.9)	3 (0.9)	5 (1.5)	2 (0.6)
Low (< 5)	17 (4.6)	6 (35.3)	11 (64.7)	5 (29.4)	8 (47.1)	1 (5.9)	2 (11.8)	0	1 (5.9)	0
**HGB (g/dl)**										
High (> 15)	16 (4.3)	7 (43.8)	9 (56.2)	7 (43.8)	3 (18.8)	0	1 (6.3)	0	0	0
Normal (9–15)	323 (86.8)	114 (35.3)	209 (64.7)	130 (40.2)	120 (37.2)	10 (3.1)	44 (13.6)	3 (0.9)	4 (1.2)	2 (0.6)
Low (< 9)	33 (8.9)	14 (42.4)	19 (57.6)	10 (30.3)	14 (42.4)	2 (6.1)	5 (15.2)	0	2 (6.1)	0
**HCT (%)**										
High (> 52)	2 (0.5)	1 (50)	1 (50)	0	1 (50)	0	0	0	0	0
Normal (30–52)	314 (84.4)	111 (35.4)	203 (64.6)	129 (41.1)	113 (35.9)	10 (3.2)	42 (13.4)	3 (0.9)	4 (1.3)	2 (0.6)
Low (< 30)	56 (15.1)	23 (41.1)	33 (58.9)	18 (32.1)	23 (41.1)	2 (3.6)	8 (14.3)	0	2 (3.6)	0
**MCV (fl.)**										
High (> 55)	38 (10.2)	13 (34.2)	25 (65.8)	14 (36.8)	19 (50)	1 (2.6)	8 (21.1)	1 (2.6)	1 (2.6)	1 (2.6)
Normal (39–55)	332 (89.2)	121 (36.4)	211 (63.6)	132 (39.8)	118 (35.5)	11 (3.3)	42 (12.7)	2 (0.6)	5 (1.5)	1 (0.3)
Low (< 39)	2 (0.5)	1 (50)	1 (50)	1 (50)	0	0	0	0	0	0
**MCH (pg)**										
High (> 17.5)	14 (3.8)	5 (35.7)	9 (64.3)	8 (57.1)	5 (35.7)	1 (7.1)	4 (28.6)	1 (7.1)	1 (7.1)	1 (7.1)
Normal (12.5–17.5)	357 (96)	129 (36.1)	228 (63.9)	139 (38.9)	132 (37)	11 (3.1)	46 (12.9)	2 (0.6)	5 (1.4)	1 (0.3)
Low (< 12.5)	1 (0.3)	1 (100)	0	0	0	0	0	0	0	0
**MCHC (g/dl)**										
High (> 36)	7 (1.9)	3 (42.9)	4 (57.1)	4 (57.1)	0	0	0	0	0	0
Normal (30–36)	289 (77.7)	105 (36.3)	184 (63.7)	114 (39.4)	103 (35.6)	12 (4.2)	36 (12.5)	3 (1.0)	6 (2.1)	2
Low (< 30)	76 (20.4)	27 (35.5)	49 (64.5)	29 (38.2)	34 (44.7)	0	14 (18.4)	0	0	0
**WBC (× 10**^**3**^**µl)**										
High (> 14)	280 (75.3)	102 (36.4)	178 (63.6)	109 (38.9)	98 (35)	10 (3.6)	34 (12.1)	1 (0.4)	5 (1.8)	1 (0.4)
Normal (5.5–14)	89 (23.9)	33 (37.1)	56 (62.9)	36 (40.4)	36 (40.4)	2 (2.2)	14 (15.7)	2 (2.2)	1 (1.1)	1 (1.1)
Low (< 5.5)	3 (0.8)	0	3 (100)	2 (66.7)	3 (100)	0	2 (66.7)	0	0	0
**PLT (× 10**^**3**^**µl)**										
High (> 800)	6 (1.6)	3 (50)	3 (50)	3 (50)	1 (16.7)	0	1 (16.7)	0	0	0
Normal (800–300)	278 (74.7)	105 (37.8)	173 (62.2)	113 (40.6)	95 (34.2)	10 (3.6)	37 (13.3)	3 (1.0)	5 (1.8)	2 (0.7)
Low (< 300)	88 (23.7)	27 (30.7)	61 (69.3)	31 (35.2)	41 (46.6)	2 (2.3)	12 (13.6)	0	1 (1.1)	0

Abbreviations: *WBC* white blood cell; *RBC* red blood cell; *HGB* hemoglobin; *HCT* hematocrit; *MCV* mean corpuscular volume; *MCH* mean corpuscular hemoglobin; *MCHC* mean corpuscular hemoglobin concentration; *PLT* platelets; *BB* *Babesia* spp; *HM* Hemoplasmas; *HP Hepatozoon* spp

*Statistically significant differences (*P* < 0.05)

### Sequence analysis

For each genus of VBP detected from the blood samples, positive amplicons were subjected to sequencing and BLAST analysis. All obtained sequence for each pathogen were found to share over 99.0% identity with reported *Candidatus* Mycoplasma haemominutum (GenBank accession numbers KR905451, KU645935), *Candidatus* Mycoplasma turicensis (GenBank accession numbers DQ464423, KR905458) and *Mycoplasma hemofelis* (Genbank accession numbers KU645929, KR905465). Regarding *Babesia* and *Hepatozoon* species, all positive amplicons share 99.7% − 100% sequence identity with isolates of *Babesia vogeli* (GenBank accession numbers MN823219) and *Hepatozoon canis* (Genbank accession numbers KU765201), respectively.

## Discussion

The presence of *Babesia, Hepatozoon* and hemotropic mycoplasma (generally called VBPs) infections in the cats from Bangkok, Thailand was demonstrated in this study. Our results showed that the most prevalent pathogens were *Babesia* and and hemoplasma whereas *Hepatozoon*. was less frequently detected. The overall prevalence of *Babesia* (39.5%; 147/372) infection in the cats in the current study was higher than the results of a previous study carried out in Thailand over a decade ago, where infection was 1.4% in a population of 1,490 cats tested [9]. Similarly, the prevalence of hemoplasmas (36.9%;137/372) infection was higher in the cats in the current research compared to recent previous studies [17], while *Hepatozoon* (3.2%;12/372) had a lower rate of the infection than the previous report [18]. The rate of *Babesia* and hemoplasmas in our study were similar to those described in other studies performed in Spain [12], Germany [19], Japan [20] and Iran [21]. In general, the differences in prevalence could have been due to several factors such as the varying nature of the cats being sampled in different studies (healthy versus sick/ hospitalized/ anemic populations/ pet versus feral), detection methods (conventional PCR with sequencing versus species-specific qPCR) and/or geographical variation [12]. The most prevalent co-infection of these VBPs in this study were with *Babesia* and hemotropic mycoplasma. It has been demonstrated that concurrent infection of *Babesia* with other hemoparasites, notably hemotropic mycoplasma species or *Hepatozoon* species, is common in endemic regions [22]. The finding regarding VBP co-infection in the current study supported this fact. Moreover, the present study was the first to confirm this important information which should enhance awareness and alert the authorities and veterinarians of the situation of VBP infection in the studied area.

Consistent with this survey, other reports have also noted that male cats and adult cats (older than 1 year) had a higher risk of hemoplasmas infection in the majority of prevalence studies carried out worldwide [12] [21]. It has been shown in several studies that male cats, especially if not neutered, have more aggressive interactions which may enhance transmission via infected blood, and adult animals have a greater chance of exposure to blood-sucking arthropods [11, 23]. Besides, the roaming cats have been reported to get more chances in close contact with wild animals which was considered as a potential source for many different infectious diseases [11]. However, our study and other investigations have failed to demonstrate the association of male or adult cats with *Babesia* infection and *Hepatozoon* infection [9].

The main and suspected vectors of *Babesia* and *Hepatozoon* are the brown dog tick, *Rhipicephalus sanguineus* and of hemoplasmas are the flea, *Ctenocephalides felis* [24], which are found in warm and temperate regions all over the world. The location chosen for sample collection in the current research at 13.7563° N, 100.501° E was in a region with relatively high temperatures and humidity year-round, with an average low temperature of 22.0 °C (https://en.wikipedia.org/wiki/Bangkok#Climate). Consequently, this climate might support the development of a high variety of blood-feeding arthropods compared to other temperate areas [25]. The overall prevalence of ectoparasites found in our study was much lower than that described in former investigation in Thailand, where 95.8% of 575 cats had ectoparasites [15]. Clearly, the campaigns of occasionally administering prophylactic vaccination and anti-ecto- and anti-endoparasitic drugs to stray dog and cat populations in this community since 2005 have had a considerable positive impact on zoonotic disease elimination and prevention [15]. The studied cats in this research were suffering from infestations with fleas, ticks and lice, of which fleas were the most common (46.5%), followed by lice (1.6%) and ticks (0.8%), respectively. This finding was in agreement with similar studies done elsewhere, as fleas have been reported to be the predominant species parasitizing cats [15]. Thailand, a tropical country, has many different blood-sucking ectoparasites; in particular, infestation with the flea *C. felis* is common in cats [17, 24] and the current results re-enforced this. The flea (*C. felis*) has always been suspected as being the natural route for hemoplasmas transmission; however, the attempts of the current study and of epidemiological study in proving the association between flea infestation and hemoplasmas infection have not been successful [12]. Interestingly, although the number of tick-infested cats was low, the prevalence of *Babesia-*infected cats in our study was large. Potentially, another route of transmission such as by blood exchange in fighting animals [26] or by vertically transplacental transmission [27] could have occurred and been involved in the spread of these pathogens in the current study.

While the most frequently described clinical signs in cats with hemoplasmosis are related to the occurrence of anemia [12, 28], the current study failed to demonstrate a statistical association between the hemoplasmas infection or any VBP infection with anemia, leukopenia, leukocytosis and thrombocytopenia. This finding was somewhat surprising but was in agreement with studies undertaken in stray cats in Italy [10] and in client-owner cats in Switzerland [29]. The clinical signs and laboratory findings for VBP infection depend on a wide range of factors. Concurrent diseases, previous infections or the species with distinct virulent of causative agents (e.g. *Mycoplasma haemofelis, Candidatus* Mycoplasma hemominutum, *Candidatus* Mycoplasma turicensis ) involved could considerably change these findings [12]. Specifically, Mhf and CMhm have been found to have the association with the existence of anemia in infected cats [12]. However, the present study has failed to identify the association between hemoplasma species and hematological changes due to the number of hemoplasma- infected samples which is representative for species identification was insufficient. Generally, younger dogs are more likely to develop clinical diseases in babesiosis and hepatozoonosis [22], yet this has been indiscernible in a cat population [22]. The level of parasitemia is usually low in cats infected with *Hepatozoon*, with less than 1% of the neutrophils containing gamonts [22]. If cats are able to tolerate more severe anemia than dogs, then the cats may act as a potential reservoir of VBPs without showing any specific clinical evidence [22, 30, 31].

An interesting outcome was the association between the laboratory hematological findings with the arthropod presence in cats. The hematological findings of the present study revealed decrease values of total erythrocyte hemoglobin concentration on the vector-borne infested cats compared with the negative group. A macrocytic hypochromic anemia represented by an increase in the MCV and a decline in the MCHC was determined in the ectoparasite-infested cat group compared to the non-infested group presented in this study. In agreement with our results, blood- sucking arthropods may play a role in the cause of blood-loss anemia in animals [32]. Ectoparasite infestation by ticks in some livestock animal has been reported to cause alterations in blood parameters including lower values for red blood cell count, packed cell volume, hemoglobin concentration and platelets count [33]. Furthermore, other factors such as nutritional status, co-infection of retrovirus (FIV, FeLV) and endoparasite infections are also known to affect hematocrit and hemoglobin levels in animals [34]. Further study is needed to improve those limitations.

## Conclusions

The results indicated a high prevalence of VBP infection circulating in cats in the study area. There was a significant reduction in the hematological parameters of cats following ectoparasite burdens. The results also revealed out that macrocytic hypochromic type of anemic condition may occur in cats infested with ectoparasites in the study area. Further studies are recommended to be carried out in different parts of the country to further establish the effects of ectoparasite infestation on the hematological and pathological parameters in cats and other domestic animals. Campaigns of VBPs monitoring in Bangkok emphasizing on the investigation of vectors and possible routes of the infection in animals should be conducted to prevent the transmission of the pathogens.

## Methods

### Study areas and animal population

Animal population were calculated according to approval standard formula as previously described [21]. This study is focused on semi-domesticated cats dwelling in 53 monasteries belonging to 34 districts in the Bangkok Metropolitan Area. In total, 372 semi-domesticated cats were sampled between March to June in 2017.

### Sample and data collection

Blood samples were collected for evaluation of hematological indicators and confirmation of VBPs was conducted using molecular method. Briefly, one milliliter of blood sample was obtained aseptically from the jugular vein of each cat and preserved in ethylene diamine tetra acetic acid (EDTA)-treated tubes. Subsequently, blood samples were used for hematological investigation and an aliquot was stored at -20 °C until further use for molecular investigation. Data collection including age (animals ≤ 12 months of age were considered juvenile, whereas all others were considered adult) and gender (male or female) were collected by interviewing from the caretakers in the monastery. Besides, all the cats were not administered anti-ectoparasite drugs before inspecting for the presence of ectoparasites (fleas, ticks or lices) by carefully combing for at least five minutes throughout its whole-body surface. All the species of ectoparasite were morphologically identified [35]. Animal ethic was approved by the Animal Ethics Committee of Kasetsart University, Bangkok, Thailand (ACKU60-VET006).

### Hematological investigation

Cell blood count (CBC) was performed within 24 hours after blood collection using a fully automatic hematology analyzer (Siemens Healthcare GmbH, Germany) at the Veterinary Medicine Teaching Hospital of Kasetsart University (Bangkok, Thailand). Hematological abnormalities in the cats were determined regarding the presence or absence of anemia (Hematocrite; HCT < 30%), leukopenia (White blood cells; WBC count < 5.5 × 103/µL), leukocytosis (WBC > 14 × 103/µL) and thrombocytopenia (Platelet; PLT < 300 × 103/µL) [15]. The red blood cell (RBC) indices consisted of the mean corpuscular volume (MCV), mean corpuscular hemoglobin (MCH) and mean corpuscular hemoglobin concentration (MCHC); these were used, whenever possible, to detect the type of anemia presenting in the infected cats. The reference interval of CBC, anemia degree and anemia classification were employed following a previous study [11].

### PCR amplification and sequence analysis

An E.Z.N.A.® Blood DNA Mini Kit (Omega Biotek Inc., Norcross, Georgia, USA) was used for the DNA extraction from the whole blood samples. The DNA was assessed for quality at 260/280 nm and quantity at 260/230 nm using a spectrophotometer (Specord plus, Analytik Jena AG, Germany). In total, 372 samples of DNA were processed using the PCR protocol based on the amplification of a specific partial sequence of the *18S* rRNA gene of *Babesia* spp. and *Hepatozoon* spp. and of the *16S* rRNA gene of feline hemotropic mycoplasmas, respectively [36–38]. The sequences of primers used and the product size of the target gene specific for each vector-borne pathogen are shown in Table 4. The reactions were performed in an automatic DNA thermal cycler MasterCycler Nexus Gradient (Eppendorf AG, Hamburg, Germany) including negative and positive controls for each sample. The PCR product was further visualized using ethidium bromide fluorescence after electrophoresis in a 1.5% agarose gel at 100 V for 40 min. Subsequently, cats with negative PCR results for VBPs were indicated as the control group and cats with positive PCR results for VBPs were considered as the positive group.

**Table 4:** Primer and protocols used for amplification of some vector-borne pathogens

Organism	Target gene	Product size (bp)	PCR primers (5’-3’)	Reference
*Babesia* spp.	18S rRNA	422–440	F: GTTTCTGMCCCATCAGCTTGA R: CAAGACAAAAGTCTGCTTGAAAC	[36]
*Hepatozoon* spp.	18S rRNA	666–800	F: ATACATGAGCAAAATCTCAAC R: CTTATTATTCCATGCAG	[37]
Hemoplasmas	16S rRNA	595–618	F: ATACGGCCCATATTCCTACG R: TGCTCCACCACTTGTTCA	[38]

For sequence analysis, selected positive amplicons were snipped from the gel and purified using FavorPrep™ GEL/PCR Purification Kit (Favorgen, Ping-Tung Agricultural Biotechnology, Ping-Tung, Taiwan). Subsequently, the purified product was submitted for Sanger DNA sequencing (Macrogen, Seoul, Korea). After obtaining the sequence results, the sequences were compared with published isolates using the Basic Local Alignment Search Tool (BLAST) of the U.S. National Center for Biotechnology Information (https://blast.ncbi.nlm.nih.gov/Blast.cgi).

### Statistical analysis

Statistical analysis of the findings obtained using PCR and the categorical variables of age, gender, presence of ectoparasite and hematological abnormalities (anemia, leukopenia, leukocytosis and thrombocytopenia) were analyzed using a chi-square test (cell frequency > 5) or Fisher’s exact test (cell frequency ≤ 5). Any parameters statistically linked to positive PCR results were used in a logistic regression model with the odds ratio (OR) to test for independent risk factors associated with infection.

Descriptive statistics (mean, median, standard deviation (SD), minimum and maximum) were obtained for the continuous variables (RBC, Hemoglobin (HGB), HCT, MCV, MCH, MCHC, WBC, PLT count value). Distribution of the data for normality was assessed using the Kolmogorov-Smirnov test and subsequently a t-test or Mann-Whitney U test, respectively, were used to test differences between the feline vector-borne pathogen positive and negative cats depending on whether the data were normally distributed or not.

Significance was established at *P* < 0.05. Both the *P* value and the OR with a 95% confidence interval (CI) were reported. Data were analyzed using the R software package for statistical analysis [39].

## Data Availability

The datasets generated and/or analysed during the current study are available in the Sequence Read Archive database in NCBI and can be accessed through accession numbers MW377729-MW377733 and MW377923-MW377924.

## Abbreviations

VBPs: Vector-borne pathogens
PCR: Polymerase chain reaction
CBC: Cell blood count
RBC: Red blood cell
WBC: White blood cell
HGB: Hemoglobin
HCT: Hematocrit
PLT: Platelet
MCV: Mean corpuscular volume
MCH: Mean corpuscular hemoglobin
MCHC: Mean corpuscular hemoglobin concentration
OR: Odd ratio
SD: Standard deviation
CI: Confident interval
FIV: Feline immunodeficiency virus
FeLV: Feline leukemia virus
